# Nursing students and depressive symptomatology: an observational study in University of Palermo

**DOI:** 10.1108/MIJ-10-2019-0006

**Published:** 2019-11-04

**Authors:** Omar Enzo Santangelo, Sandro Provenzano, Domiziana Giordano, Enrico Alagna, Francesco Armetta, Claudia Gliubizzi, Antonio Terranova, Giuseppe D'Anna, Dalila Barresi, Dimple Grigis, Cristina Genovese, Raffaele Squeri, Alberto Firenze

**Affiliations:** University of Palermo, Palermo, Italy; University Hospital “P. Giaccone”, Palermo, Italy; University of Palermo, Palermo, Italy; University of Bergamo, Bergamo, Italy; University of Messina, Messina, Italy; University of Palermo, Palermo, Italy

**Keywords:** University, Depression, Female gender, Health status, Mood disorders, Nursing students

## Abstract

**Purpose:**

Depression is a common and serious medical illness, considered as a public health issue because it interferes with the interpersonal, social and professional functioning of the individual, and its frequency is constantly increasing. According to a recent review, approximately 34 per cent of nursing students had experienced depression worldwide. The university period may represent a moment in which the mental well-being of students is subjected to stress with a relative predisposition to the development of diseases related to mood disorders. The purpose of this study is to estimate the prevalence and examine the socio-demographic correlates of depressive symptomatology.

**Design/methodology/approach:**

In April 2019, a questionnaire was administered to all the nursing students of University of Palermo of the three years of course, accompanied by informed consent. Multivariable logistic regression was performed. The statistical significance level chosen for all analyses was 0.05. The results were analyzed using the STATA statistical software version 14. Results are expressed as adjusted odds ratio (aOR) with 95 per cent confidence intervals.

**Findings:**

The sample consists of 493 students who completed the questionnaire, and the average age of the sample participants is 21.88 years. The multivariable logistic regression model shows that the risk to have depressive symptomatology is significantly associated with the following independent variables: female gender (aOR 1.91), being single (aOR 1.87), second year of study (aOR 1.94), third year of study (aOR 1.92), not performing regular physical activity (aOR 1.78) and perceived low health status (aOR 3.08).

**Originality/value:**

This study shows that belonging to the female gender, being further along in the years of study, having a chronic illness and perceiving a low state of health are all factors that can increase the risk of developing the symptoms of depression; rather, regular physical activity, friendship and romantic relationships can be considered factors protecting them from the risk of falling into depression that can undermine both the study and work performance. Certainly, it is important to analyze all the involved variables to improve the global health not only of the nursing students but of all the students.

## Introduction

Depression (major depressive disorder) is a common and serious medical illness, considered a public health issue. It interferes with the interpersonal, social and professional functioning of the individual and causes feelings of sadness and loss of energy, initiative and interest in activities once enjoyed. Furthermore, depression may lead to feeling worthless or guilty, difficulty in thinking, concentrating or making decisions, lack of self-care, changes in appetite (weight loss or gain unrelated to dieting), slowed movements and speech, insomnia or hypersomnia and thoughts of death or suicide. Depression is the most common mental disorder in the general population, with a frequency between 3 and 5 per cent, according to the World Health Organization (WHO), and this frequency is steadily increasing. The aggregate point, one-year and lifetime prevalence of depression are 12.9, 7.2 and 10.8 per cent, respectively (Lim *et al.*, 2018). The Epidemiologic Catchment Area Study, driven by National Institute of Mental Health, has estimated an annual incidence of major depression of 1.10 per cent in men and 1.98 per cent in women. According to WHO, by 2020, major depression will be at second place, after ischemic coronaropathy, for disability adjusted life years, that represents the lost years of healthy life, both for premature death and for disability (WHO, 2009; Murray and Lopez, 1997). Depression, indeed, is strongly linked with higher rates of morbidity, mortality and also suicide. This disorder can affect all ages, even if the age at which the first symptoms appear is commonly around 15-29 years old (Chen *et al.*, 2019). The university period, in particular, may represent a moment in which the mental well-being of students is subjected to stress with relative predisposition to the development of diseases related to mood disorders (Santangelo *et al.*, 2018).

Indeed, the university brings big changes in to students’ life. The pace of life becomes more intense, the workload of studies is increased, and often the geographical distance of the family may provoke feelings such as disappointment, irritability, anxiety and impatience during graduation (Fernandes *et al.*, 2018; Ibrahim *et al.*, 2013; Provenzano *et al.*, 2018; Santangelo *et al.*, 2019; Santangelo *et al.*, 2018). Such situations are, in many cases, anxiety factors and possible triggers for depression. A high prevalence of depression in university students is observed, on average 30.6 per cent, while for the population in general, this prevalence corresponds to 9 per cent (Fernandes *et al.*, 2018). In particular, when compared with other university students, nursing students have additional factors that may cause or contribute to depressive symptoms, such as disinterest in the nurse course, a low-grade point average, worry about future placement, workload, study assignments, fear of unknown conditions, handling technical equipment, mistakes with patients in clinical practices and situations in which they must deal with the imminence of death (Chen *et al.*, 2019; Fernandes *et al.*, 2018). Many studies have confirmed that nursing students have a high risk of depressive symptoms and depression. According to a recent review, approximately 34.0 per cent of nursing students had experienced depression worldwide (Tung *et al.*, 2018). Depressive disorders can lead to the onset of a reduction of academic achievement and abuse of alcoholic beverages (Eisenberg *et al.*, 2009). Moreover, studies have shown that depression is among the strongest risk factors for suicide attempt in nursing students (Aradilla-Herrero *et al.*, 2014). Data from longitudinal studies show that these symptoms persist for a long period of time if students do not receive appropriate help (Newbury-Birch *et al.*, 2002). Therefore, it is necessary to identify valid and protracted approaches that aim to help nursing students deal with depressive symptoms and depression.

## Objectives

The objective of our study was to estimate the prevalence and examine the socio-demographic correlates of depressive symptomatology within the student population of the degree course in nursing at the University of Palermo to focus attention on the mental and behavioral disorders of this population, to improve the quality of life and to prevent the future development of diseases.

## Materials and methods

This study was approved by the Ethical Committee of the University Hospital “P. Giaccone” of Palermo, Minutes No. 02/2019 (15. Studio A.D.A.3) of February 18, 2019. In the month of April 2019, a survey was provided to all the nursing students of University of Palermo of the three years of course, accompanied by informed consent. In the first section of the questionnaire, personal information was requested, relating to the course of study undertaken, job, chronic illnesses, the perception of the economic and health status and voluptuous habits. In the second part of the survey, the Quick Inventory of Depressive Symptomatology Self-Report (QIDS-SR16) questionnaire was administered, a self-report tool that allows evaluation of the severity of depressive symptomatology by administering 16 items with 4 possible answers to which a score ranging from 0 to 3 is attributed. The QIDS-SR16 is derived from the 30-item Inventory of Depressive Symptomatology, which has seen many years of use at the University of Texas Southwestern Medical School (Rush *et al.*, 2003).

Questions in the QIDS-SR16 include: sleep disturbance (initial, middle and late insomnia or hypersomnia), sad mood, decrease/increase in appetite/weight, concentration, self-criticism, suicidal ideation, interest, energy/fatigue and psychomotor agitation/retardation. Based on the score, the subjects are assigned to one of the following categories: (0-5) no depressive symptomatology, (6-10) mild, (11-15) moderate, (16-20) severe and (≥21) very severe depressive symptomatology.

The variable “age” was subsequently dichotomized in <22 years and ≥22 years because the average age of the sample participants was 21.88 years. For all qualitative variables, absolute and relative frequencies have been calculated; categorical variables were analyzed by Pearson’s chi-square test (*x*^2^). Multivariable logistic regression was performed, considering it as a dependent variable “depressive symptomatology moderate-severe-very severe.” To evaluate the role of the variables in the first section of the questionnaire, the covariates to be included into the final model were selected using a stepwise backward selection process, with a univariate *p*-value <0.25 as the main criterion (Hosmer and Lemeshow, 1989). The statistical significance level chosen for all analyses was 0.05. The results were analyzed using the STATA statistical software version 14 (StataCorp, 2015). Results are expressed as adjusted odds ratio (aOR) with 95 per cent confidence intervals (CI).

## Results

The sample size consists of 493 students who agreed to the informed consent and completed the questionnaire. Only five third-year students did not complete the questionnaire, two of them refused consent and three were not traceable. The average age of the sample participants is 21.88 years (standard deviation ± 3.38), 67.55 per cent of the interviewees are women, 99.59 per cent were born in Italy, 71.60 per cent are single, 40.57 per cent report to attend the first year of study, 30.83 per cent attend the second year of study and 28.60 are in the third year of study. In all 39.55 per cent were off-site students, 78.50 per cent live with their families, 7.30 per cent report that they currently have a job, 77.28 per cent report a low perceived economic status, 29.41 per cent currently smoke, 59.23 per cent do not exercise regularly, 26.57 per cent report a low perceived health status and 11.77 per cent of the interviewees show a moderate severe anxiety symptomatology (Table I). Regarding bivariate analysis, statistically significant differences were found for the following variables: “gender?”, “are you a student off-site or in-site or commuter students?”, “do you perform regular physical activity?”, “perceived health status”, and “do you have chronic illnesses?” (Table II). Table III shows aOR. A multivariable logistic regression model was used based on 493 observations. Each independent variable has been adjusted for all the other independent variables. The analysis shows that the risk to have depressive symptomatology (moderate, severe or very severe) is significantly associated with the following independent variables: female gender (aOR 1.91, 95 per cent CI 1.05-3.48, *p* = 0.035); being single at the time of study (aOR 1.87, 95 per cent CI 1.02-3.44, *p* = 0.044); second year of study (aOR 1.94, 95 per cent CI 1.03-3.65, *p* = 0.042); third year of study (aOR 1.92, 95 per cent CI 1.03-3.59, *p* = 0.040); not performing regular physical activity (aOR 1.78, 95 per cent CI 1.02-3.11, *p* = 0.044) and perceived low health status (aOR 3.08, 95 per cent CI 1.80-5.25, *p* < 0.001).

## Discussion

“What a stress!” “What anxiety!” “What a depression!” The boys often use these exclamations, in many cases as a simple interlayer, in others as a real expression of emotional malaise. In Italy there are about 800,000 depressed young people: an international survey conducted by Sodexo (2019), a company that deals with services aimed at improving the quality of life, shows that more than 4,000 young Italian university students are most dissatisfied with their lives. Among the main concerns of young people are the workload and too many commitments (Johnson *et al.*, 2018). More and more young people manifest personality disorders, lack of attention, listlessness, anxiety, depression and obsessive manias, and the phenomenon unfortunately seems to be increasing in recent years. The depressive symptomatology among students today represents an extremely important problem that can influence and compromise both academic performance and also work performance (Santangelo *et al.*, 2018). Juvenile depression is constantly growing and it is assumed that in 2030, it will be the most widespread chronic disease among the very young population (Crouch *et al.*, 2019; Werner-Seidler *et al.*, 2017). A very important fact that makes one think: why are young people so depressed? Unhappiness in young people is the consequence of the contemporary lifestyle. From our study conducted on a total of 493 students, of which 32.45 per cent are males (160), 67.55 per cent are females (333) and 40.16 per cent (198) are younger than 22 years and 59.84 per cent (295) are over the age of 22, it emerges that female individuals suffer from depressive symptoms more than male subjects. In fact, according to the data emerging from the multivariate logistic regression (Table III), female individuals have a 1.91 times greater risk than male subjects of being suffering from depressive disorder (moderate, severe or really severe). In adolescence and in adulthood, major depressive disorder occurs more commonly in women, with a ratio of 2:1 compared to males (APA [American Psychological Association], 2019). The female preponderance in depression seems to begin around puberty, suggesting a probable link of the disorder to the woman’s generative period. Serretti *et al.* (2004) also confirms this fact, citing the fact that numerous studies have revealed a greater involvement in depressive states, especially between the ages of 15 and 30. As shown in the literature (Pulkki-Råback *et al.*, 2012), being single at the time of study predisposes to a greater risk of depressive symptomatology (aOR 1.87). The data present in the literature (Chernomas and Shapiro, 2013), however, leave many possibilities for interpretation open. For example, according to a hypothesis provided by a team of researchers, friendships and romantic relationships would provide relief from stress and increase the level of self-esteem of children, guaranteeing the development of effective interpersonal skills. The direct result of this would be the increase in the coping skills of young people in difficult situations (Provenzano *et al.*, 2018). Years of university studies can upset the mental well-being of students, making them vulnerable to the damaging effects of many stressors, in many cases reducing academic performance, as reported in literature and academic sources (Chernomas and Shapiro, 2013). Students later in the study years, second year (aOR 1.94) and third year (aOR 1.92), have a greater risk of developing severe and very severe depressive symptoms. Performing regular physical activity decreases the risk of suffering from depression (aOR 1.78). One aspect that is not immediately thought of when talking about the benefits of exercise is its link with mental health. Moving in fact helps to prevent depression too. It is an aspect that should not be underestimated and that makes the movement an important tool both in a preventive way and for the treatment of depressive states (Atkins, 2017). The WHO recalls the benefits of physical activity on whole well-being, mind and body, which recommends 150 min of physical activity per week, aerobic and mild to moderate, for adults up to 65 years. Now new evidence on the close link between movement and mental well-being comes from an international study which was also attended by the University of New South Wales in Sydney (Australia) and published in the *American Journal of Psychiatry*. Regular exercise, at any intensity, has been associated with a reduction in the risk of depression (Andersson *et al.*, 2015). The researchers collected data on physical activity levels and depressive symptoms in 33.908 Norwegian adults. It was found that 12 per cent of cases of depression could have been prevented if participants had only taken an hour of physical activity a week. In addition, those who reported being sedentary were 44 per cent more likely to develop depression than those who said they would engage in physical activity one to two hours a week. With physical exercise the body releases a series of neurotransmitters, first of all the endorphins, which are associated with a feeling of well-being and are activated by brain mechanisms modulated by physical activity (Italian Ministry of Health, 2019). Finally, individuals who perceive a lower health status have a greater risk of developing depressive symptoms (aOR 3.08). Depression is a condition associated with suffering and disability and constitutes a significant source of direct and indirect costs. In adolescence, the presence of depressive symptomatology is a frequent and often serious condition, which can be associated with a low perception of the status of health, socio-economic status or the presence of diseases (Han *et al.*, 2019).

## Conclusions

Systematic review and meta-analysis show that a high-pooled prevalence of depression was reported among nursing students (Tung *et al.*, 2018). Sleep disturbance (initial, middle and late insomnia or hypersomnia), sad mood, decrease/increase in appetite/weight, concentration, self-criticism and psychomotor agitation/retardation until suicidal ideation are only some of the sensations characterizing depressive status, with a different weight depending on the severity, that we tried to analyze. Our study, carried out on nursing students of the University of Palermo, also tried to identify the copious variables associated with this status, variables that have to be known to intervene and prevent the important consequences that can manifest in young people who are depressed. Being female, further ahead in the year of study, having chronic illness and perceiving low health status are factors that can increase the risk to develop depressive symptoms. On the contrary, performing regular physical activity, friendship and romantic relations can be helpful to face with more positivity the problems and difficulties that young students can bump into during their academic career, protecting them from the risk of falling into depression that can undermine both study and work performances, with direct and indirect costs. Certainly it is important to be able to recognize all the signs that can reveal a suffering mental status, to not confuse a serious psychiatric syndrome, as depression is, with a simple bad mood. This would enable us to analyze all the variables involved to improve the global health not only of the nursing students, but also of the nurses that they would become and, as consequence, of their patients. In conclusion, this study shows that the high prevalence of depression among the students can be mitigated only with continual efforts by the education institutions and further research.

## Acknowledgement

*Funding*: None.

Competing interests: None declared.

*Ethical approval:* This study was approved by the Ethical Committee of the University Hospital “P. Giaccone” of Palermo, Minutes No. 02/2019 (15. Studio A.D.A.3) of February 18, 2019.

*Author’s contribution statement:* Sandro Provenzano, Omar E. Santangelo, Domiziana Giordano and Alberto Firenze conceived, designed, coordinated and supervised the research project. Sandro Provenzano and Omar E. Santangelo performed the data quality control, optimized the informatics database, performed the statistical analyses and evaluated the results. Sandro Provenzano, Omar E. Santangelo, Domiziana Giordano, Enrico Alagna, Francesco Armetta, Claudia Gliubizzi, Dalila Barresi, Dimple Grigis, Alberto Firenze, Antonio Terranova, Giuseppe D’Anna, Cristina Genovese and Raffaele Squeri wrote the manuscript. All authors read and approved the final manuscript.

*Submission declaration:* The submission has not been previously published.

## Figures and Tables

**Table I:** Description of the sample

**Variables**	*N*	(%)
***Gender***
Male	160	32.45
**Female**	333	67.55
***Age class***		
< 22 years old	198	40.16
**≥ 22 years old**	295	59.84
***Country of birth***		
Italy	491	99.59
**Other**	2	0.41
***Are you engaged or single?***		
Engaged	140	28.40
**Single**	353	71.60
***Year of study***		
First	200	40.57
**Second**	152	30.83
**Third**	141	28.60
*Are you a student off-site or in-site or commuter students?*		
In-site	183	37.12
**Commuter student**	115	23.33
**Off-site**	195	39.55
*Do you live with your family?*		
Yes	387	78.50
**No**	106	21.50
*Do you have a job right now?*		
No	457	92.70
**Yes**	36	7.30
*Perceived economic status*		
Medium-high	112	22.72
**Low**	381	77.28
*Do you currently smoke?*		
No	348	70.59
**Yes**	145	29.41
*Do you perform regular physical activity?*		
Yes	201	40.77
**No**	292	59.23
*Perceived health status*		
Medium-high	362	73.43
**Low**	131	26.57
*Do you have chronic illnesses?*		
No	463	93.91
**Yes**	30	6.09
*Depressive symptomatology*		
None	271	54.97
**Mild**	140	28.40
**Moderate**	64	12.98
**Severe**	17	3.45
**Very severe**	1	0.20
*Age*		
21.88 (SD ± 3.38*)		

Note: *Mean and standard deviation

**Table II:** Bivariate associations between the depressive symptomatology and the variables of the first section of questionnaire

	Depressive symptomatology					
**Variables**	None (%)	Mild (%)	Moderate (%)	Severe (%)	Very severe (%)	*p*-v*alue*
*Gender*
Male	107 (66.88)	36 (22.50)	11 (6.88)	6 (3.75)	0 (0.00)	0.003
**Female**	164 (49.25)	104 (31.23)	53 (15.92)	11 (3.30)	1 (0.30)	
*Age class*							
≥ 22 years old	114 (57.88)	53 (26.77)	25 (12.63)	5 (2.53)	1 (0.51)	0.548
**< 22 years old**	157 (53.22)	87 (29.49)	39 (13.22)	12 (4.07)	0 (0.00)	
*Country of birth*							
Italy	270 (54.99)	140 (28.51)	63 (12.83)	17 (3.46)	1 (0.20)	0.597
**Other**	1 (50.00)	0 (0.00)	1 (50.00)	0 (0.00)	0 (0.00)	
***Are you engaged or single?***							
Engaged	83 (59.29)	40 (28.57)	16 (11.43)	1 (0.71)	0 (0.00)	0.226
**Single**	188 (53.26)	100 (28.33)	48 (13.60)	16 (4.53)	1 (0.28)	
***Year of study***							
First	113 (56.50)	62 (31.00)	19 (9.50)	6 (3.00)	0 (0.00)	0.463
**Second**	80 (52.63)	44 (28.95)	22 (14.47)	6 (3.95)	0 (0.00)	
**Third**	78 (55.32)	34 (24.11)	23 (16.31)	5 (3.55)	1 (0.71)	
***Are you a student off-site or in-site or commuter students?***							
In-site	101 (55.19)	53 (28.96)	24 (13.11)	5 (2.73)	0 (0.00)	0.072
**Commuter student**	51 (44.35)	41 (35.65)	15 (13.04)	8 (6.96)	0 (0.00)	
**Off-site**	119 (61.03)	46 (23.59)	25 (12.82)	4 (2.05)	1 (0.51)	
***Do you live with your family?***							
Yes	217 (56.07)	104 (26.87)	51 (13.18)	14 (5.62)	1 (0.26)	0.672
**No**	54 (50.94)	36 (33.96)	13 (12.26)	3 (2.83)	0 (0.00)	
***Do you have a job right now?***							
No	253 (55.36)	126 (27.57)	62 (13.57)	15 (3.28)	1 (0.22)	0.419
**Yes**	18 (50.00)	14 (38.89)	2 (5.56)	2 (5.56)	0 (0.00)	
***Perceived economic status***							
Medium-high	70 (62.50)	28 (25.00)	12 (10.71)	2 (1.79)	0 (0.00)	0.391
**Low**	201 (52.76)	112 (29.40)	52 (13.65)	15 (3.94)	1 (0.26)	
***Do you currently smoke?***							
No	187 (53.74)	104 (29.89)	44 (12.64)	12 (3.45)	1 (0.29)	0.778
**Yes**	84 (57.93)	36 (24.83)	20(13.79)	5 (3.45)	0 (0.00)	
***Do you perform regular physical activity?***							
Yes	125 (62.19)	54 (26.87)	18 (8.96)	4 (1.99)	0 (0.00)	0.033
**No**	146 (50.00)	86 (29.45)	46 (15.75)	13 (4.45)	1 (0.34)	
*Perceived health status*							
Medium-high	225 (62.15)	95 (26.24)	35 (9.67)	7 (1.93)	0 (0.00)	<0.001
**Low**	46 (35.11)	45 (34.35)	29 (22.14)	10 (7.63)	1 (0.76)	
***Do you have chronic illnesses?***							
No	258 (55.72)	135 (29.16)	57 (12.31)	12 (2.59)	1 (0.22)	<0.001
**Yes**	13 (43.33)	5 (16.67)	7(23.33)	5 (16.67)	0 (0.00)	

Note: Used Pearson’s chi-square test

**Table III:** Multivariable logistic regression

	Depressive symptomatology (moderate-severe-very severe)	
**Independent Variables**	aOR (95% CI)	*p*-v*alue*
***Gender***
Male	1	
**Female**	1.91 (1.05-3.48)	0.035
***Are you engaged or single?***		
Engaged	1	
**Single**	1.87 (1.02-3.44)	0.044
***Year of study***		
First	1	
**Second**	1.94 (1.03-3.65)	0.042
**Third**	1.92 (1.03-3.59)	0.040
***Do you perform regular physical activity?***		
Yes	1	
**No**	1.78 (1.02-3.11)	0.044
***Perceived health status***		
Medium-high	1	
**Low**	3.08 (1.80-5.25)	<0.001
***Do you have chronic illnesses?***		
No	1	
**Yes**	2.20 (0.94-5.16)	0.070

Notes: Stepwise backward selection process with a univariate *p*-value < 0.25 as the main criterion. aOR are presented. Each independent variable is adjusted for all the other independent variables. Based on 493 observations
